# Multi-centre Study of Vaping Effects on COVID-19 Presentations in Western Sydney Australia

**DOI:** 10.7759/cureus.63190

**Published:** 2024-06-26

**Authors:** Anne Lazari, Daniel Sada

**Affiliations:** 1 Medicine, Oceania University of Medicine, Sydney, AUS; 2 Family Medicine, Greenway Medical Hub, Sydney, AUS

**Keywords:** electronic cigarettes (e-cigarettes), effects of vaping, vaping-related lung injury, vaping and covid-19, covid-19

## Abstract

Aim*:* This preliminary pilot study aimed to explore a correlation between COVID-19 presentation with e-cigarette vapers in the Western Sydney region of Australia.

Method: Extracted data from questionnaires completed by 100 Australians residing in the Western Sydney region who were infected with COVID-19 during the pandemic from March 2020 to March 2022 were analyzed. Data collected included patient age, biological gender, years of vaping, if vaping began prior to COVID-19, weekly vaping frequency, the year that COVID-19 was contracted, and the category of COVID-19 symptoms. The symptoms are scaled into four categories for this study, from lowest severity to high severity based on the World Health Organization’s classifications.

Results: Vapers in Western Sydney Australia experienced higher severity COVID-19 presentations compared to non-vapers. Biological males were found to be more susceptible than biological females for experiencing the highest severity. Vapers with higher years of vaping had severe COVID-19 presentations compared to lesser years of vaping.

Conclusion: Among adults in Western Sydney Australia aged 18 years and above, the collected data showed a correlation between e-cigarette vapers reporting higher severity of COVID-19 presentations compared to non-vapers of the same demographic when they contracted COVID-19.

## Introduction

Prior to the emergence of COVID-19, Australia witnessed an increase in vaping by almost four times with a number of users aged 18-24 years old from 6.8% in 2016 to 18.7% in 2019. Furthermore, a recent study found people aged 14 and over using e-cigarettes had tripled from 1.1% in 2019 to 3.5% in 2022-2023 [1]. The statistics are assumed greater due to the illegal online sales that are not regulated in Australia, especially during the COVID-19 lockdowns. According to the Australian COVID-19 statistics [2], the transmission of COVID-19 in Australia was the highest in the state of Sydney, specifically the Western region, which coincidently was the highest state for vaping; therefore, the focus for this study is based in this region. The Western Sydney region is known to be more socioeconomically disadvantaged compared to Metropolitan Sydney [3], which could be a considerable factor impacting the increase in vaping behaviour and COVID-19 infections and worsened clinical presentations/symptoms (herein symptoms/clinical presentations were used interchangeably). The changed attitude towards vaping caused an increase in usage kickstarted by COVID-19 lockdowns for two primary reasons: boredom and a substitute for traditional tobacco cigarettes [4].

E-cigarettes, also known as vapes, were made by a Chinese pharmacist Hon Lik in 2003 [5]. E-cigarettes are a type of electronic atomizing cigarette that gained popularity as a potential cessation device or an alternative to traditional tobacco cigarettes. E-cigarettes are also known as electronic nicotine delivery systems (ENDS) or electronic non-nicotine delivery systems (ENNDs) [6]. The components of these devices include a power source (usually lithium) to heat the liquid in the cartridge, an atomizer that vaporizes the liquid, an e-liquid solution containing 4-(methylnitrosamino)-1-(3-pyridyl)-1-butanone (NNK) and N′- nitrosonornicotine (NNN), tetrahydrocannabinol (THC), vitamin E acetate additive [7], and flavoring such as bubble gum, mint, and butter, which specifically contain diacetyl. Other components included such as propylene glycol and vegetable glycerin are used to retain moisture. The emissions from e-cigarettes were found to be formaldehyde and acrolein, which are both known carcinogens causing toxicity [8].

Furthermore, these vaping devices can include nicotine components, which in Australia is illegal under the therapeutic goods administration (TGA) regulator guidelines, but with online or illegal sales, vapes with nicotine have been used by Australians. Notably, in Australia, nicotine inclusion in vaping devices is approved under the provision of medical use only as endorsed by a registered Australian physician and approved by the TGA. For this study, it is reasonable to assume that the data collected are of e-cigarettes with no nicotine addiction.

Considering the harmful components of the devices, there is growing evidence that e-cigarettes cause respiratory tract toxicity, including oral, nasal, and pulmonary [8,9], which is shown to weaken the immunity response putting users at greater risks of viral and bacterial, and for this study in particular, we are exploring the effects of vaping on COVID-19 virus [10] and any related complications particularly acute lung injury that required hospital admissions [11].

In August 2019, a new diagnosis for lung-related injury [11] due to vaping was introduced by the Centers for Disease Control and Prevention known as e-cigarettes or vaping use-associated lung injury (EVALI) [12,13]. Roughly 3,000 cases and 68 deaths were reported across the U.S. from EVALI [14]. This new respiratory illness diagnosis associated with vaping supports the need for this study to explore if individuals who vaped experienced worsened COVID-19 presentations [15] or if the potential risks of e-cigarettes are overstated [16].

COVID-19 disease emerged in December 2019 following an outbreak in China that spread globally caused the COVID-19 pandemic. The symptoms and clinical presentations of COVID-19 varied from mild respiratory illness and recovery without special treatment to severe respiratory stress and/or hospitalizations. According to the World Meter, a tracker for global disease statistics, there were 6,504,981 global deaths caused by COVID-19 (data recorded 06 September 2022). COVID-19’s mode of transmission is via small liquid particles by coughing, sneezing, speaking, singing, or breathing. As a result, vaping may not only worsen COVID-19 symptoms but also influence COVID-19 transmission amongst young people through coughing whilst vaping or sharing devices [17].

While uncertainty remains regarding the correlation between vapers and worsened COVID-19 presentations [18], calls for concerns by health professionals in 2022 led the Australian government to propose stronger regulation and enforcement of all e-cigarettes with an allocated budget of $63 million set for public health information campaigns to discourage Australians from taking up vaping, as well as smoking traditional tobacco cigarettes [19].

The symptoms of COVID-19 vary in commonality according to the World Health Organization (WHO); for the purpose of this study, the symptoms will be based on WHO categorization and scaled into four categories from lowest severity (fever, cough, tiredness, loss of taste or smell) to highest severity (hospital admissions); the latter added for the purpose of this study to explore complications are associated with vaping and COVID-19 infection (as discussed in the Method section). It is known people with underlying conditions such as cardiovascular illnesses, diabetes, chronic respiratory diseases, and cancer are known to have a higher risk of developing more severe symptoms when infected with COVID-19 [20]; for this reason, these diseases serve as the exclusion criteria for this study (as discussed in the Method section).

To the best of our knowledge, there is limited research available to show a correlation between vaping and the severity of COVID-19 clinical presentation during the pandemic, hence the need for our research exploring this area.

## Materials and methods

Study design and material

As this is a preliminary small pilot study, the data extracted are from questionnaires completed by 100 Australians residing in the Western Sydney region who were infected with COVID-19 during the pandemic from March 2020 to March 2022 and were used for analysis to explore any associations. Of the 100-sample size, 50 were confirmed vapers, and 50 were non-vapers serving as control for this study. Data collected included patient age, biological gender, years of vaping, vaping prior to COVID-19 vaping frequency, year(s) of COVID-19 infection, four categories of COVID-19 presentations, and vaccination status. The questionnaire was designed with nine questions (Appendix 1).

Materials required are in the form of a questionnaire. COVID-19 presentations/symptoms (herein presentations/symptoms are interchangeable) were scaled into four categories; the most common (lowest severity: category one) symptoms were fever, cough, tiredness, and loss of taste or smell. The less common (lower severity: category two) symptoms were sore throat, headache, aches, pains, diarrhea, a rash on the skin, discoloration of fingers or toes, and red or irritated eyes. The severe illness (high severity: category three) symptoms were difficulty breathing or shortness of breath, loss of speech or mobility or confusion, and chest pain. The complicated (extreme severity: category four) symptoms were any hospital admissions related to COVID-19 infection. These categories are influenced by WHO categorization of COVID-19: most common: fever, cough, tiredness, and loss of taste or smell; less common: sore throat, headache, aches and pains, diarrhea, a rash on the skin, discoloration of fingers or toes, and red or irritated eyes; and serious symptoms: difficulty breathing or shortness of breath, loss of speech or mobility or confusion, and chest pain.

Questionnaires were collected from multiple centers in the Western Sydney area from November 2022 to December 2022. No names, addresses dates of birth, or any other identifiers of participants were presented in the questionaries.

The names and addresses of the medical centers that were used to distribute the questionaries were Greenway Medical Hub Suite 101, Level 1/1183-1187 The Horsley Drive, Wetherill Park NSW Sydney 2164, High Performance Health Unit 101c/1187 The Horsley Dr, Wetherill Park NSW 2164, and Greenacre Medical Centre, 168 Waterloo Road Greenacre NSW Sydney 2190. Greenway Medical Hub and Greenacre Medical Centre are primary care centres, and High Performance Health is a physiotherapy clinic.

Inclusion and exclusion criteria

Inclusion criteria included age 18 years and above, residents of Wester Sydney region, vapers from at least 2019, regular vapers (regular vapers at least one full vaping device a week), and the year(s) COVID-19 infection occurred. Exclusion criteria included vapers with known respiratory conditions (e.g., chronic obstructive pulmonary disease (COPD), asthma, or others), vapers with comorbidities (e.g., hypertension, hypercholesterolemia, diabetes, or others), and vapers who also smoked traditional cigarettes.

Training

Reception staff in the medical centers were trained to ensure that questionnaires were completed correctly and patients were assisted by the receptionist to answer questions if needed. This ensured clean data collection for the study with 50 study samples and 50 controls.

Statistical analysis

Questionnaires were collected as manual papers, and the data were entered into Excel (Microsoft® Corp., Redmond, WA) software for analysis. The distribution of data points and a comparison of metric values across different subgroups of the data were analyzed on Excel software. For this preliminary small pilot study, the descriptive analysis method was implemented, with a summarization of data and visualized results as a bar chart to show patterns for comparison and discussion. 

Data security

The Excel document was password-locked.

Governance and ethics

All participants gave verbal consent before they participated in the study. The study was conducted in accordance with protocols of the Oceania University of Medicine and approval granted by the ethics committee of the Oceania University of Medicine.

## Results

A total of 100 participants met the inclusion criteria for the study and were included in the analysis. Sample characteristic are shown in Table 1.

**Table 1:** Sample characteristics N/A: Not applicable

Characteristic	Whole sample		Vaper		Non-vapers	
	Total 100		Total 50		Total 50	
	N	%	N	%	N	%
18-24 years	41	41	21	42	20	40
25-35 years	32	32	18	36	14	28
Equal or greater than 36 years	27	27	11	22	16	32
Biological males	52	52	31	62	21	42
Biological females	48	48	19	38	29	58
Years vaping						
<1 year	N/A	N/A	5	10	N/A	N/A
1-2 years	N/A	N/A	28	56	N/A	N/A
Equal or greater than 3 years	N/A	N/A	17	34	N/A	N/A
Frequency of vapes per week						
1	N/A	N/A	17	34	N/A	N/A
2	N/A	N/A	10	20	N/A	N/A
3	N/A	N/A	19	38	N/A	N/A
4+	N/A	N/A	4	8	N/A	N/A
COVID-19 symptoms						
Category 1	21	21	6	12	15	30
Category 2	38	38	14	28	24	48
Category 3	32	32	23	46	9	18
Category 4	9	9	7	14	2	4
Vaccinated for COVID-19						
No	69	69	29	58	40	80
Yes	31	31	21	42	10	20

**Figure 1:**
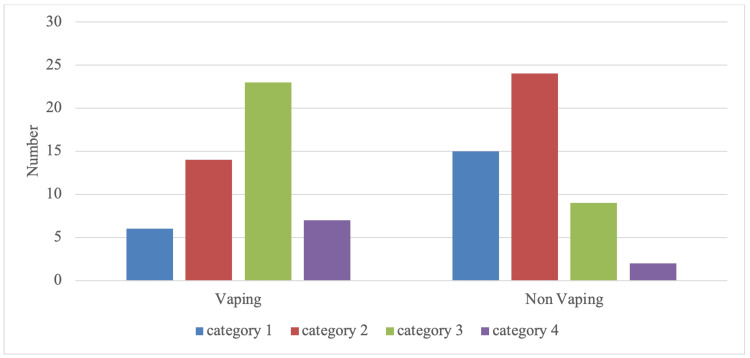
Vaping vs non-vaping association with COVID-19 symptoms

Of the total 100 sample size, two groups (vapers and non-vapers) were compared based on the COVID-19 symptoms experienced in the four categories, with vapers showing a significant increase in category three and four COVID-19 symptoms compared to non-vapers, whilst category one and two showed higher prevalence in non-vapers (Figure 1).

**Figure 2:**
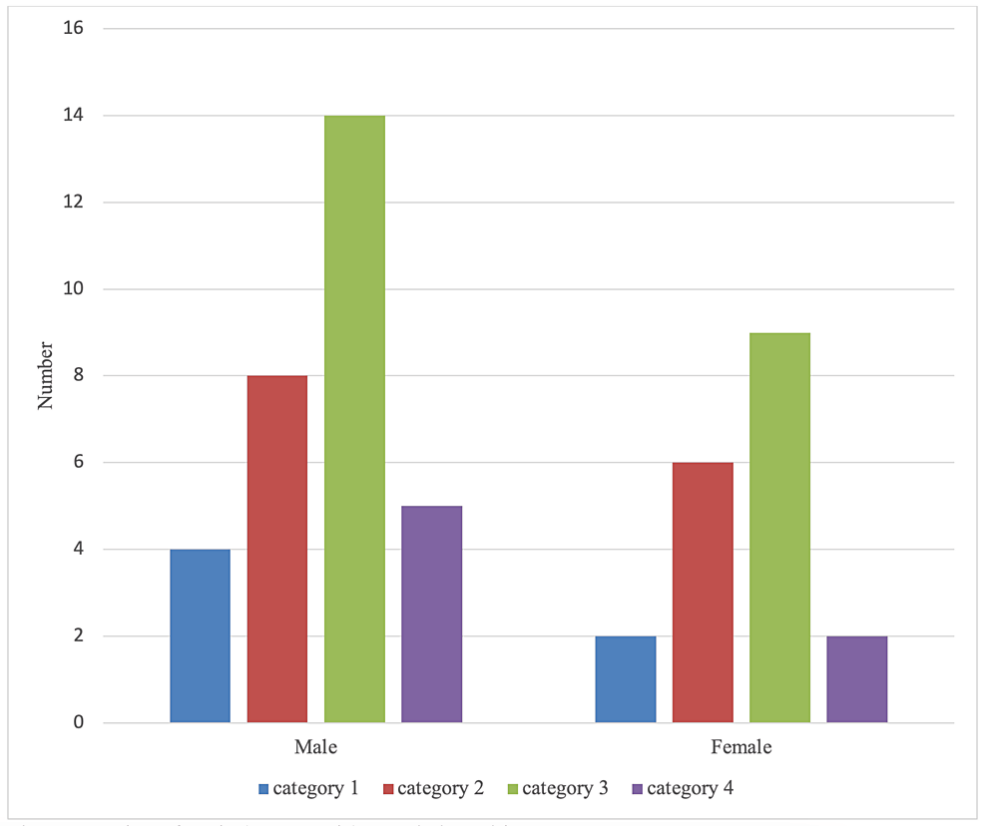
Male vs female (vapers only) association with COVID-19 symptoms

There were 31 male vapers (N = 31, 62%) compared to 19 (N =19, 38%) female vapers. Considering that almost double of the vapers were males, the male vapers showed a more significant increase in category three in comparison to the female vapers (Figure 2). When adjusted for the difference in gender vaping sample size (numbers normalized), females experienced slightly higher category three symptoms compared to males, whilst the males experienced significantly higher category four symptoms compared to females (graph not shown).

**Figure 3:**
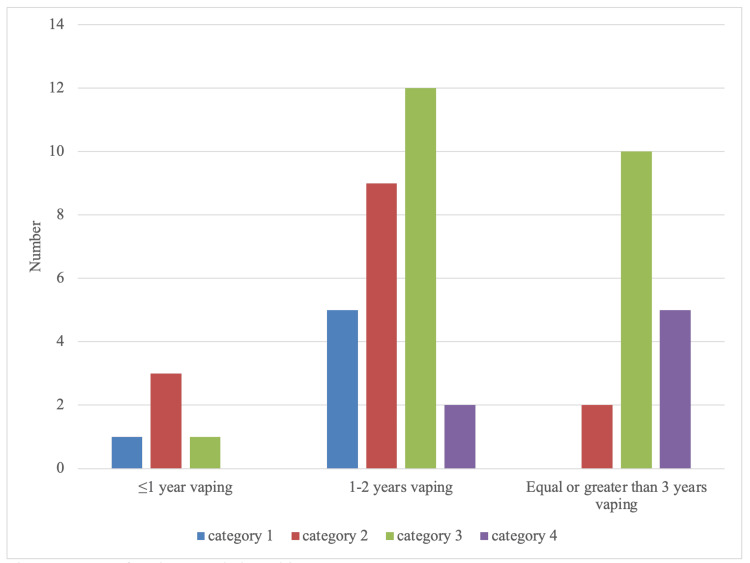
Years of vaping association with COVID-19 symptoms

The number of years vaped between one and two years by both males and females showed a notable increase in category three COVID-19 presentations. More vapers experienced complications in category four when they vaped for three years or more (Figure 3). The graph overall shows a higher ratio of more severe symptoms as the number of years of vaping increases.

**Figure 4:**
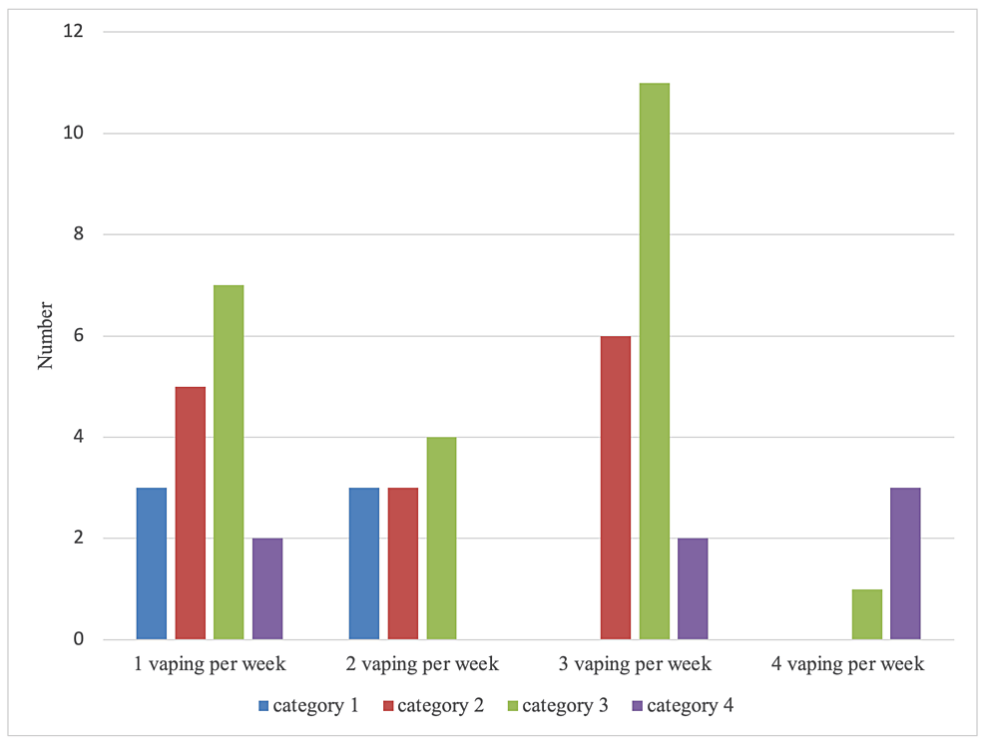
Vaping frequency association with COVID-19 symptoms

Males and females who vaped three containers per week showed a substantially higher incidence in category three presentations compared to the other categories. Moreover vaping four times a week showed a concentrated number of subjects experiencing category four symptoms and the absence of lower-category symptoms. The other categories did not show a significant comparison (Figure 4).

**Figure 5:**
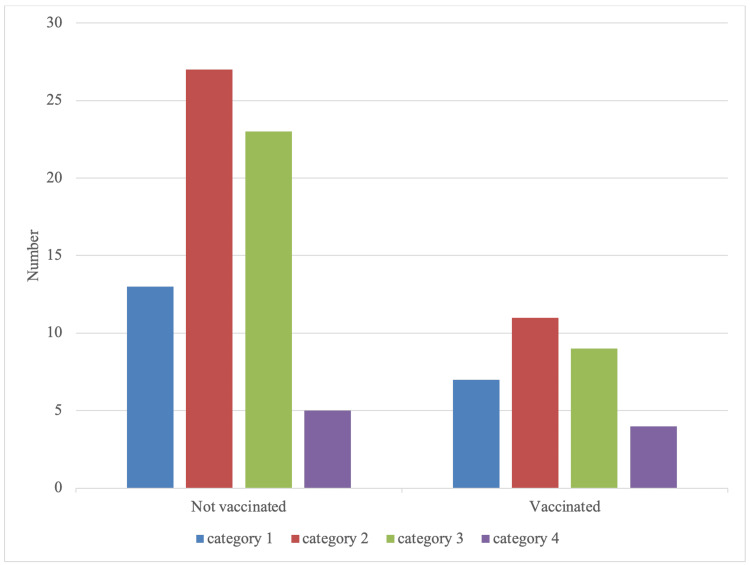
Vapers (vaccinated vs non-vaccinated) association with COVID-19 symptoms

There were 69 (N = 69, 69%) non-vaccinated compared to 31 (N = 31, 31%) vaccinated subjects. Male and female vapers who were vaccinated experienced an overall lower COVID-19 presentations in all four categories compared to the vaccinated group. When adjusted for differences in sample size in each group, the non-vaccinated still had a higher prevalence in categories two and three compared to the vaccinated, while in category four, there was a slight increase in the vaccinated compared to the non-vaccinated. Category three and Category four showed a more significant increase between the groups in comparison to categories one and four (Figure 5).

## Discussion

To the best of our knowledge, this small preliminary study is among the few available in Australia investigating the effects of vaping with COVID-19 clinical presentations. The sample of 100 Western Sydney Australians showed an association of more severe COVID-19 presentations (difficulty breathing or shortness of breath, loss of speech or mobility or confusion, chest pain, and reports of more hospital admissions) for the 50 vaping group compared to the 50 non-vaping control group.

Considering the reports on respiratory injuries and the decrease in immunity capabilities [21,22] consequential to the use of e-cigarettes, the data collected demonstrated a trend of the effects of vaping on the respiratory system. Possible causes for worsening symptoms include the aerosolization of flavoring compounds of e-cigarette liquids, tetrahydrocannabinol-based oils or vitamin E, or the illegal use of nicotine in vaping containers [23]. The severity of symptoms showed an increase as the number of vaping years increased and as the number of vapes used per week increased possibly due to prolonged damage of lung and related respiratory tissue with more, repeated exposure to the toxins. Furthermore, if vaping is found to be damaging to lung tissue, the reoccurrence of vaping episodes could in theory prevent the healing process, compounding the damage dealt to the subject over time.

While vaping is likely less toxic than smoking cigarettes given the lack of combustible tobacco constituents, there is uncertainty about the health hazard of produced toxins when they are vaporized in the container [24]. Notably, heating the mix in the vaping device generates toxins that are delivered at variable doses by the vaping technique producing a spectrum of respiratory tissue responses such as cough, dyspnea, chest pain, and fatigue [25] due to the increased inflammatory and oxidative stress biomarkers and suppressed immune responses, which can see vapers put at a higher risk for experiencing worsened COVID-19 symptoms.

The study showed that more males are susceptible than females to experiencing category three severe symptoms (such as difficulty breathing or shortness of breath, loss of speech or mobility or confusion, and chest pain) and category four symptoms, which was any hospital admission related to COVID-19 infection. Similar research was conducted [26] where males were found to experience more vaping complications than females. Possible causes could be due to vaping techniques and/or behaviors, meaning that males may more likely believe that vaping made for men is more socially accepted compared to females’ who may believe that society disapproves of vaping behavior for females [27].

Of the 50 sample sizes of female and male vapers, those vaccinated were affected to a lesser extent by COVID-19 severity of symptoms. These results agree with previous studies [28] for the benefits of vaccination for reducing COVID-19 symptoms [29]. This study did not analyze or compare the different COVID-19 vaccines available in the Australian market; the data focused on demonstrating a COVID-19 vaccination regimen were beneficial to the vaping group.

The strength of this study includes the randomized assignment of participants into two groups, which helped eliminate selection bias in the study sample. Secondly, the exclusion criteria eliminating comorbidities and risk factors of a history of traditional cigarette smoking increased the likelihood of producing reliable results and eliminating potentially substantial confounders known to exacerbate outcomes of COVID-19 infection [30].

Data collected in this study were subjected to at least six limitations. First, the sample size was not significantly large to indicate a strong increase in worsened COVID-19 symptoms. This small study laid a stepping foundation for further larger studies to include a wider Australian population. Moreover, a change in location in Australia (compared to Western Sydney only) could likely include a substantially different demographic, which could bring into question genetic differences in vaping tolerance and/or COVID-19 symptoms. Second, the data collected in 2023 required questionnaire participants to recall symptoms from three years ago, which could have been subject to recall bias. Third, e-cigarette use could have contained additional substances to their cartilages that are outside of the approved manufacturer content, which could have implicated respiratory injury and consequent symptoms. Fourth, long-term follow-ups could show the long-term effects of vaping on the respiratory system. Fifth, there is variability in vaccines in terms of conferred COVID-19 protection (via neutralizing antibodies), as well as the length of protection provided, particularly when accounting for decreasing COVID-19 antibody titers in the blood over time. Sixth, as vaping devices are sold in different-sized containers, the effects of vaping could be influenced by the concentration of vaping products introduced to the body, depending on whether the container is larger or smaller.

Furthermore, whilst not a limitation of this study, the findings were based on a quantitative analysis (via questionnaire) of subjects. To further build on this, quantitative studies assessing the levels of nicotine or other vaping product markers in the body against the severity of symptoms could further add weight and potentially corroborate this study’s findings of vaping’s effects on COVID-19 severity.

## Conclusions

The objective of this small preliminary pilot study was to explore if vaping worsened COVID-19 presentations when the virus was contracted by Australians who vaped during the pandemic in the Western Sydney region. This study's findings support an association whereby males and females who vaped were more likely to experience more severe COVID-19 symptoms and overall worsened COVID-19 presentations.

We recommend more in-depth research that is needed to further assess risks of vaping long term on the body’s immune response to viruses such as COVID-19 that are known to target the respiratory system.

## Appendices

This is a medical research survey on vaping status and COVID-19 presentations for Sydney residents aged over 18 years old. This medical research is conducted by Anne Lazari, a medical student at the Oceania University of Medicine. Your name and other identifiers are not required or used. By filling out this form, you provide consent. If you have any further questions, please contact anne.lazari@oum.edu.ws

**Table 2:** Questionnaire

Question (Q)			Answer			
Q1		What is your age?	□ 18-24 years	□ 25-35 years	□ Equal or greater than 36 years	
Q2		What is your biological gender?	□ Biological Female	□ Biological Male		
Q3	a)	Do you vape?	□ Yes	□ No		
	b)	Were you vaping at least 1 year before the COVID-19 pandemic was declared in Australia, in March 2020?	□ Yes	□ No		
	c)	How many years have you been vaping?	□ ≤ 1 year	□ 1-2 years	□ ≥ 3 years	
	d)	Frequency of vapes per week? (i.e., containers used)	□ 1	□ 2	□ 3	□ 4+
Q4		Did you get COVID-19? If yes, which year?	□ No	□ Yes		
				□ 2020	□ 2021	□ 2022
Q5		What were your COVID-19 symptoms?	Category 1: □ Fever □ Cough □ Tiredness □ Loss of taste or smell □ Diarrhea Category 2: □ Sore throat □ Headache □ Aches and pains □ Diarrhea □ Rash on skin □ Discoloration of fingers or toes □ Red or irritated eyes Category 3: □ Difficulty breathing □ Shortness of breath □ Loss of speech or mobility □ Chest pain Category 4: □ Yes Hospital Admission			
Q6		Were you vaccinated for COVID-19?	□ Yes	□ No		
Q7		Do you or have you ever smoked cigarettes?	□ Yes	□ No		
Q8		How many cigarettes do/did you smoke a day and for how many years?				
Q9		Please list any medical conditions you may have. For example: diabetes, hypertension, asthma, respiratory conditions.	Write none if no conditions			
